# An Unusual Presentation of Philadelphia Chromosome Positive B‐Cell Acute Lymphoblastic Leukemia With Isolated Osteolytic Lesions at Diagnosis: A Case Report

**DOI:** 10.1155/crh/2728902

**Published:** 2026-07-17

**Authors:** Tiantian Zhang, Omer Jamy, Sravanti Rangaraju

**Affiliations:** ^1^ Department of Internal Medicine, University of Central Florida/HCA, Pensacola, Florida, USA; ^2^ Department of Medicine, Division of Hematology/Oncology, University of Alabama at Birmingham, Birmingham, Alabama, USA, uab.edu

**Keywords:** acute lymphoblastic leukemia (ALL), bone marrow, osteolytic lesion, Ph+ B-ALL, tyrosine kinase inhibitors

## Abstract

**Background:**

Philadelphia chromosome–positive B‐cell acute lymphoblastic leukemia (Ph + B‐ALL) is a high‐risk subtype of ALL characterized by the presence of the BCR‐ABL1 fusion gene and typically presents with diffuse bone marrow involvement, circulating blasts, and systemic symptoms. While extramedullary disease may occur, it is rarely the dominant or sole feature at diagnosis. Isolated skeletal involvement with a normal bone marrow evaluation is exceedingly rare and diagnostically challenging.

**Case Presentation:**

We report the case of a 56‐year‐old Caucasian female who presented with progressive left hip pain and extensive lytic skeletal lesions, initially raising concern for multiple myeloma or metastatic cancer. Imaging revealed multifocal FDG‐avid lesions in the skeleton without evidence of a systemic disease. Bone biopsy confirmed precursor B‐lymphoblastic leukemia with expression of CD45, CD34, CD79a, TdT, and CD99, and eventually detection of BCR::ABL1 p190 transcript, consistent with Ph + B‐ALL. Surprisingly, bone marrow aspirate and biopsy showed normocellular trilineage hematopoiesis without morphologic or immunophenotypic evidence of leukemia. The patient was treated with hyperfractionated cyclophosphamide, vincristine, doxorubicin, and dexamethasone alternating with high dose methotrexate and cytarabine (HyperCVAD/MA) plus dasatinib followed by allogeneic stem cell transplantation, achieving complete molecular and metabolic remission.

**Conclusion:**

This case highlights an atypical presentation of Ph + B‐ALL with isolated extramedullary skeletal involvement and a normal bone marrow at diagnosis. Such presentations, although rare, underscore the importance of maintaining a broad differential diagnosis in patients with unexplained lytic bone lesions and unremarkable hematologic findings. Early biopsy and appropriate molecular testing of affected tissue are essential for timely diagnosis and initiation of appropriate targeted therapy.

## 1. Background

Acute lymphoblastic leukemia (ALL) is an aggressive hematologic malignancy characterized by clonal proliferation of immature lymphoid precursors in the bone marrow, peripheral blood, and occasionally extramedullary tissues. It accounts for approximately 20% of adult leukemias and remains the most common childhood cancer, although the prognosis and therapeutic approaches differ significantly between age groups. In adults, approximately 75% of ALL cases are of B‐cell lineage, with the remainder arising from T‐cell precursors [1]. Compared to pediatric cases, adult ALL has historically been associated with lower remission rates and poorer long‐term survival.

Among its subtypes, Ph + B‐ALL represents approximately 20% of adult ALL cases [2]. The hallmark of Ph + ALL is the presence of the BCR‐ABL1 fusion gene, resulting from a reciprocal translocation between Chromosomes 9 and 22 [t (9; 22)(q34; q11)], which encodes a constitutively active tyrosine kinase that drives leukemogenesis [3].

Clinically, ALL often presents with signs of bone marrow failure, such as fatigue, fever, bleeding, and infections. Bone pain is another common symptom and is often a result of rapid marrow expansion and leukemic infiltration. However, direct skeletal involvement, such as cortical bone destruction, osteolytic lesions, or periosteal reaction, is rare in adults and may radiographically mimic primary bone tumors or metastatic disease [4, 5]. Diagnosis of ALL requires bone marrow aspiration and biopsy, which typically reveal hypercellularity with a predominance of lymphoblasts. Flow cytometry, cytogenetic analysis, and molecular testing are essential to confirm lineage, identify the presence of BCR‐ABL1, and guide targeted therapy [6].

The advent of tyrosine kinase inhibitors (TKIs) such as imatinib, dasatinib, and ponatinib has dramatically altered the therapeutic landscape for Ph + ALL. When combined with chemotherapy regimens or immunotherapy such as blinatumomab, TKIs have significantly improved remission rates and overall survival [7–9].

The true incidence of isolated osteolytic Ph + ALL remains unknown. This case report presents a patient with Ph + B‐ALL who exhibited atypical isolated skeletal involvement at diagnosis, emphasizing the diagnostic challenges, the role of targeted therapy, and the importance of early molecular characterization in guiding personalized treatment approaches, as well as highlights the curative potential of allogeneic hematopoetic stem cell transplant (HSCT) in extramedullary Ph + ALL.

## 2. Case Presentation

A 56‐year‐old Caucasian female with a past medical history of eczema, uterine fibroids, telangiectasias, and osteopenia on Vitamin D repletion was referred for assessment of suspected multiple myeloma versus metastatic disease of unknown origin, given extensive osseous involvement. The patient reported left hip pain persisting for over a year, with significant worsening in the preceding 6 months after a fall. She denied constitutional symptoms, cough, shortness of breath, other bone pain, headaches, dizziness, nausea, vomiting, abdominal pain, or changes in bowel habits. Baseline laboratory findings are summarized in Table 1.

**TABLE 1:** Selected laboratory and molecular findings at diagnosis.

Category	Laboratory/finding	Result
CBC	WBC	6.10 × 10^3^/μL
	Absolute neutrophils	4.41 × 10^3^/μL
	Hemoglobin	12.8 g/dL
	Platelets	236 × 10^3^/μL

CMP/chemistry	Creatinine	0.8 mg/dL
	ALT	18 U/L
	AST	18 U/L
	Alkaline phosphatase	43 U/L
	LDH	162 U/L

Coagulation	PT	13.9 s
	INR	1.08
	Fibrinogen	415 mg/dL

Molecular testing	Blood BCR‐ABL PCR	Undetected
	BCR‐ABL PCR from iliac/hip lesion	100%

Imaging	PET finding at diagnosis	Multifocal areas of abnormal FDG uptake throughout the skeleton

*Note:* FDG, fluorodeoxyglucose; LDH, lactate dehydrogenase.

Abbreviations: CBC, complete blood count; CMP, comprehensive metabolic panel; INR, international normalized ratio; PET, positron emission tomography; PT, prothrombin time.

Initial imaging with CT of the abdomen and pelvis revealed lytic lesions in the left acetabulum and proximal right femoral diaphysis, as well as a suspected lytic lesion in the proximal left femur. A pathological fracture was noted in the left acetabulum. No evidence of a primary malignancy was identified in the chest, abdomen, or pelvis. MRI of the hips confirmed these findings and revealed a femoral lesion at risk for impending pathological fracture. The patient underwent biopsy of the bone lesion and found to have B‐ALL.

Bone marrow aspiration and biopsy were performed for further characterization and interestingly revealed normocellular marrow (30%–40%) with trilineage hematopoiesis, no overt dysplasia, and no morphologic or immunophenotypic evidence of B‐lymphoblastic leukemia. Flow cytometry of the bone marrow demonstrated a small B‐lineage compartment (0.7% of total cells), consisting of CD19 + CD10+ precursor B‐cells (0.1%) and polyclonal mature B‐cells (0.6%). Peripheral blood flow cytometry demonstrated no evidence of a significant immature myeloid population (< 0.01% of total cells), with a predominance of granulocytes (69.2%) and monocytes (7.4%). BCR::ABL PCR in the bone marrow and peripheral blood was less than 0.1%. Corresponding fluorescent in situ hybridization (FISH) for t (9; 22) (q34; q11) was negative in the bone marrow. PET imaging showed multifocal FDG‐avid lesions throughout the skeleton, including the proximal right femoral diaphysis, left inferior pubic ramus, left acetabulum, left ilium, right scapula, and left anterior second rib. No hypermetabolic lymphadenopathy or extraosseous disease was identified (Figure 1).

**FIGURE 1:**
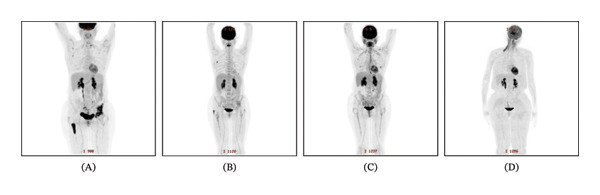
Positron emission tomography scans during diagnosis, treatment response, and follow‐up. (A) Initial positron emission tomography (PET) scan demonstrating multifocal areas of abnormal fluorodeoxyglucose (FDG) uptake throughout the skeleton. The most intense activity is observed in the proximal right femoral diaphysis, with additional hypermetabolic lesions involving the left inferior pubic ramus, left acetabulum, left ilium, right scapula, and left anterior second rib. (B) Interval imaging shows a marked decrease in metabolic activity in the proximal right femoral marrow lesion and the mixed lytic‐sclerotic lesion of the left iliac‐pubic region. No new aggressive osseous lesions are identified. Mild curvilinear tracer uptake overlying the greater trochanters, more prominent on the right, is likely degenerative or enthesopathic in origin. Overall findings suggest a partial treatment response. (C) Follow‐up PET demonstrates a persistent mixed lytic‐sclerotic lesion in the left ilium without significant FDG uptake, consistent with the treated residual structural change rather than metabolically active disease. No new areas of increased tracer activity are seen. (D) Previously identified osseous lesions show no abnormal radiotracer uptake above background levels, indicating disease remission. Abbreviations: FDG, fluorodeoxyglucose; PET, positron emission tomography.

Due to the lack of adequate and appropriately handled bone tissue for molecular studies, a repeat biopsy of the bone lesion was attempted this time from the iliac lesions with the assistance of Musculoskeletal Interventional Radiology. In view of the unusual bone presentation and broad differential diagnosis at the time of tissue evaluation, flow cytometry was performed using an expanded immunophenotypic panel. This identified approximately 74.2% of total cells as aberrant precursor B‐cells, characterized by CD19 positivity, heterogeneous CD10 expression, CD34 positivity, CD20 negativity, low CD9, low CD38, CD58 positivity, and low/negative CD15. In this context, low/negative CD15 did not suggest mixed phenotype leukemia but rather contributed to immunophenotypic characterization of the lesion. BCR::ABL PCR was performed on cell suspension from the bone lesion by suspending the cells in normal saline medium and revealed a BCR::ABL p190 transcript at 100% confirming Ph + B‐ALL. Corresponding FISH was positive for t (9; 22) (q34; q11) in the bone lesion.

The patient started treatment with Hyper CVAD/MA plus dasatinib and achieved complete remission after completion of HyperCVAD 1B. PCR analysis showed undetectable BCR::ABL1 transcripts in the peripheral blood, and PET imaging demonstrated a complete metabolic response although residual treated osseous structural changes persisted on subsequent imaging without abnormal FDG uptake. The patient underwent an allogeneic matched unrelated donor (MUD) stem cell transplant after Cycle 3 of chemotherapy without immediate complications (Figure 2). Graft‐versus‐host disease (GVHD) prophylaxis included tacrolimus and methotrexate. Dasatinib was resumed posttransplant at 50 mg daily per institutional protocol and continued for 5 months. It was subsequently discontinued because of bilateral lower extremity edema. A PET scan at 1 year post‐HSCT was negative for disease recurrence. The patient is currently being treated for chronic GVHD involving the liver, oral mucosa, joints/fascia, and skin, manifested by lichen planus–like lesions of the bilateral oral mucosa, 3+ pitting edema of the bilateral lower extremities from the feet to the waist, and superficial, nonhidebound, pinchable skin changes of the abdomen and bilateral flanks. Based on NIH criteria and clinical judgment, the chronic GVHD was assessed as moderate to severe. Management initially included prednisone at 1 mg/kg with taper over 8 weeks, followed by ruxolitinib 10 mg twice daily beginning approximately 9 months posttransplant and continuing until approximately 3 years posttransplant. She later received rituximab 375 mg/m^2^ weekly for 4 weeks at approximately 3 years and 8 months posttransplant, followed by axatilimab starting approximately 4 years and 2 months posttransplant, which remains ongoing at the time of this report. The patient perspective described here was obtained through direct communication during follow‐up and confirmed again at the time of consent for publication. From the patient’s perspective, the treatment course was intensive but acceptable given the goal of long‐term cure, and she expressed satisfaction with the outcome. The patient remains in complete remission at nearly 5 years from diagnosis (Figure 1).

**FIGURE 2:**
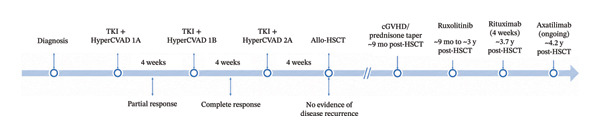
Timeline of diagnosis, treatment, response, and follow‐up. Abbreviations: allo‐HSCT, allogeneic hematopoietic stem cell transplantation; cGVHD, chronic graft‐versus‐host disease; HyperCVAD/MA, hyperfractionated cyclophosphamide, vincristine, doxorubicin, and dexamethasone alternating with high‐dose methotrexate and cytarabine; TKI, tyrosine kinase inhibitor.

The patient was treated with a TKI plus HyperCVAD/MA, achieved partial metabolic response after TKI + HyperCVAD 1A, complete molecular and metabolic response after TKI + HyperCVAD 1B, underwent allogeneic hematopoietic stem cell transplantation (allo‐HSCT) after TKI + HyperCVAD 2A, and remained without evidence of disease recurrence on follow‐up. The posttransplant course was complicated by chronic GVHD requiring systemic therapy.

## 3. Discussion

Ph + B‐ALL is a high‐risk subtype of B‐ALL where the molecular alteration drives leukemogenesis via a constitutively active tyrosine kinase and is most commonly associated with diffuse bone marrow involvement, peripheral blood blastemia, and systemic symptoms. Extramedullary involvement, while occasionally observed, is usually concurrent with marrow disease. In contrast, this case represents a strikingly atypical presentation with Ph + B‐ALL manifesting with extensive skeletal disease and no morphologic or immunophenotypic evidence of marrow involvement at diagnosis.

As presented in this case, B‐ALL has been reported to cause lytic bone lesions primarily through the aberrant expression of receptor activator of nuclear factor κB ligand (RANKL) by leukemic blasts. RANKL promotes osteoclastogenesis and bone resorption, leading to increased osteoclast activity, trabecular bone loss, and in some cases, destruction of the metaphyseal growth plate. Notably, this osteolytic process occurs independently of stromal or osteoblastic cells and can be attenuated by RANKL antagonists, underscoring the pivotal role of the RANK–RANKL axis in B‐ALL–associated bone pathology [10, 11]. Additional contributory mechanisms include the secretion of proinflammatory cytokines, such as tumor necrosis factor‐alpha (TNF‐α) and interleukin‐6 (IL‐6), which further enhance osteoclast differentiation and activity, exacerbating bone destruction [12]. Rarely, leukemic blasts may produce parathyroid hormone–related peptide (PTHrP), directly stimulating bone resorption and contributing to hypercalcemia [13]. Although extramedullary disease has been described in other BCR::ABL1‐positive leukemias, particularly CML, where extramedullary blast crisis may occur even in the setting of non–blast‐phase marrow and has been reported in a case with the e1a2/p190 transcript, there is currently insufficient evidence to conclude that p190 BCR::ABL1 in Ph + ALL specifically predisposes to isolated skeletal or other extramedullary presentation at diagnosis [14–16].

The presentation of hematologic malignancies with isolated bone involvement and normal bone marrow findings is rare but increasingly recognized in the case‐based literature. Among such entities, primary bone lymphoma, particularly diffuse large B‐cell lymphoma, is the most well‐documented, characterized by lymphoma restricted to osseous structures without evidence of systemic or marrow disease at diagnosis [17, 18]. Similarly, isolated myeloid sarcoma, also known as granulocytic sarcoma or chloroma, may manifest as a bone‐based tumor mass without concurrent marrow infiltration by acute myeloid leukemia, requiring histopathologic and immunophenotypic confirmation for accurate diagnosis [19, 20]. Hairy cell leukemia, although typically involving marrow and spleen, has been reported in exceedingly rare cases to present with isolated skeletal lesions in the absence of marrow or splenic disease [21]. Avascular necrosis of the femoral head, including in the setting of antiphospholipid syndrome, may also enter the differential diagnosis of focal hip pain and femoral abnormalities. However, the multifocal FDG‐avid osteolytic lesions and diagnostic tissue biopsy in our patient were not consistent with a non‐neoplastic ischemic process [22]. Notably, B‐ALL has not been reported as presenting with isolated extramedullary bone lesions, a normal hemogram, and no detectable bone marrow involvement at the time of initial diagnosis.

Although osteolytic involvement in B‐ALL is recognized in pediatric patients, only a handful of adult cases have been documented, and all have shown clear bone marrow disease at presentation. For example, Faiyoumi et al. described a case of B‐ALL first manifesting as diffuse lytic skeletal lesions with hypercalcemia, radiographically mimicking multiple myeloma, but subsequent marrow evaluation demonstrated 36% precursor B‐cell blasts confirming overt leukemia [23]. Likewise, El‐Ashwah et al. reported two adult patients with Philadelphia chromosome–negative B‐ALL who presented with symptomatic, disseminated osteolytic bone lesions, and marked hypercalcemia; in both cases, bone marrow aspiration revealed > 90% lymphoblasts, and treatment proceeded with standard induction chemotherapy following correction of calcium levels [24]. Importantly, while these reports highlight that B‐ALL can occasionally present with striking skeletal destruction and metabolic disturbances, they differ fundamentally from our case that every described adult patient exhibited substantial leukemic marrow infiltration at diagnosis, and most were Philadelphia chromosome–negative.

In our case, the presentation of B‐ALL with isolated skeletal involvement and no detectable bone marrow disease likely reflects an early or localized phase of leukemic infiltration, in which malignant lymphoblasts preferentially migrate to and proliferate within the bone or periosteal niche prior to involving the marrow more diffusely. This pattern may be driven by specific interactions between leukemic cells and components of the bone microenvironment, including dysregulated chemokine signaling, increased production of osteoclast‐activating factors, and localized remodeling that fosters leukemic cell survival and growth. Importantly, such atypical presentations may occur despite normal peripheral blood counts and the absence of circulating blasts, highlighting the critical role of bone marrow and early tissue biopsy in evaluating patients with unexplained lytic bone lesions and/or hypercalcemia [25, 26].

These unusual presentations highlight the diagnostic challenges when typical marrow findings are absent. In such scenarios, a high index of clinical suspicion, guided by PET imaging and confirmed by histologic and molecular analysis of affected bone lesions, is crucial to prevent misdiagnosis or treatment delay. Recognition of such patterns is not only diagnostically important but also has direct implications for therapeutic planning and prognosis [27, 28]. This case also highlights the need for general awareness on the part of treating oncologists that special sample processing may be needed when handling bone tissue for molecular studies. Appropriate sample yield is only feasible with close coordination between a multidisciplinary team of pathologists, interventional radiologist, and the treating oncologist.

This report has several limitations. As a single case, it cannot establish the incidence, underlying biology, or optimal management of isolated skeletal Ph + B‐ALL. In addition, because the initial bone biopsy specimen was not processed optimally for molecular studies, repeat tissue sampling was needed to confirm BCR::ABL1 status, which may not always be practical in routine care. The biologic basis for this unusual bone‐predominant presentation also remains speculative, as no functional studies of the leukemic microenvironment were performed. Finally, although this patient had an excellent long‐term outcome, that result should be interpreted cautiously and may not be generalizable to other patients with similar presentations.

This case also highlights several areas for future research, including better characterization of the biologic mechanisms underlying bone‐predominant or extramedullary presentations of B‐ALL, optimization of tissue‐based molecular workflows when marrow studies are nondiagnostic, and clarification of the optimal treatment approach for patients with isolated extramedullary Ph + disease.

In summary, this case illustrates a rare presentation of Ph + B‐ALL with isolated extramedullary skeletal involvement and a normal bone marrow at diagnosis. It emphasizes the importance of maintaining a broad differential diagnosis in patients with atypical osseous lesions and normal marrow workup. Early histologic sampling and molecular characterization are essential in such cases to avoid diagnostic delay and to guide risk‐adapted therapy, especially in genetically defined high‐risk subtypes such as Ph + ALL. It also highlights that these patients may achieve long‐term remission and potentially be cured with intensive chemotherapy and consolidative allogeneic stem cell transplantation. However, the role of chemotherapy free regimens in patients with isolated extramedullary disease remains unknown. As newer diagnostic technologies and targeted therapies evolve, the recognition of such uncommon disease presentations will be critical for optimizing patient outcomes.

**Nomenclature**
ALL: Acute lymphoblastic leukemia
allo‐HSCT: Allogeneic hematopoietic stem cell transplantation
ALT: Alanine aminotransferase
AST: Aspartate aminotransferase
B‐ALL: B‐cell acute lymphoblastic leukemia
BCR‐ABL1: Breakpoint cluster region–Abelson murine leukemia viral Oncogene 1
CBC: Complete blood count
CD: Cluster of differentiation
cGVHD: Chronic graft‐versus‐host disease
CML: Chronic myeloid leukemia
CMP: Comprehensive metabolic panel
CT: Computed tomography
FDG: Fluorodeoxyglucose
FISH: Fluorescence in situ hybridization
GVHD: Graft‐versus‐host disease
HSCT: Hematopoietic stem cell transplantation
HyperCVAD/MA: Hyperfractionated cyclophosphamide, vincristine, doxorubicin, and dexamethasone alternating with high‐dose methotrexate and cytarabine
IL‐6: Interleukin‐6
INR: International normalized ratio
LDH: Lactate dehydrogenase
MRI: Magnetic resonance imaging
MUD: Matched unrelated donor
NIH: National Institutes of Health
PCR: Polymerase chain reaction
PET: Positron emission tomography
Ph+: Philadelphia chromosome–positive
PT: Prothrombin time
PTHrP: Parathyroid hormone–related peptide
RANKL: Receptor activator of nuclear factor κB ligand
TdT: Terminal deoxynucleotidyl transferase
TKI: Tyrosine kinase inhibitor
TNF‐α: Tumor necrosis factor‐alpha
WBC: White blood cell

## Author Contributions

Sravanti Rangaraju and Omer Jamy were the treating physicians of this patient. Tiantian Zhang and Sravanti Rangaraju drafted the manuscript; Tiantian Zhang, Omer Jamy, and Sravanti Rangaraju critically revised and modified the manuscript. Dr. Sravanti Rangaraju had full access to all of the data in this study and takes complete responsibility for the integrity of the data and the accuracy of the data analysis.

## Funding

The authors received no funding regarding the publication of this article.

## Disclosure

All authors have read and approved the final version of the manuscript.

## Ethics Statement

Ethics approval is not required for this type of study.

## Consent

Written consent to participate in this study was obtained from the patient. Written informed consent was obtained from the patient for publication of this case report and any accompanying images. A copy of the written consent is available for review by the Editor‐in‐Chief of this journal.

## Conflicts of Interest

The authors declare no conflicts of interest.

## Data Availability

All data generated during this study can be accessed through direct communication with the corresponding author and the agreement of all research team members.
